# A rule for predicting the new equilibrated carbon dioxide partial pressure after changes in the ventilation frequency

**DOI:** 10.1186/cc10732

**Published:** 2012-03-20

**Authors:** S Buehler, M Jensen, S Lozano, S Schumann, J Guttmann

**Affiliations:** 1 Uniklinik Freiburg, Germany

## Introduction

In mechanical ventilation the arterial carbon dioxide partial pressure (PCO_2_) is one of the key parameters to control the ventilation frequency. Qualitatively, the effect of changes in the ventilation frequency on the arterial PCO_2 _level is well known. However, little is known about the time it takes for the PCO_2 _value to reach a new equilibrium after a change in the ventilation frequency (the period of latency), nor in what way the transition between two states of equilibrium takes place.

## Methods

We carried out a clinical study on patients without any history of lung disease or intracranial surgery in order to determine these relations. We collected data for the arterial PCO_2 _from blood gas analyses at discrete points in time as well as continuous end-tidal CO_2 _(etPCO_2_) and transcutaneous CO_2 _(PtcCO_2_) data and checked for the accuracy of the latter two. Least-squares fitting and a statistical analysis were carried out.

## Results

We determined a general rule to estimate the period of latency after a change in the ventilation frequency. Furthermore, we specified the relation between a change in the ventilation frequency and the change in the PCO_2 _level. Last, the transition between two PCO_2 _levels was found to follow an exponential law and the fitting resulted in a formula for the prediction of the new PCO_2 _level. The new equilibrium can be predicted with high confidence in all cases after only 3 to 4 minutes using four data points while the period of latency lasts much longer, usually between 10 and 20 minutes.

## Conclusion

The general rule for the period of latency allows an estimation of the amount of time it takes for the PCO_2 _value to stabilise again after a disturbance. A quantitative knowledge of the transition between two PCO_2 _equilibria allows for the prediction of the new PCO_2 _level long before the period of latency is over. Thus, with our relation between the change in ventilation frequency and the change in PCO_2 _at hand, an optimal PCO_2 _level can be aimed for at bedside in the shortest time span possible.

